# *Cauliflower mosaic virus* P6 Dysfunctions Histone Deacetylase HD2C to Promote Virus Infection

**DOI:** 10.3390/cells10092278

**Published:** 2021-09-01

**Authors:** Shun Li, Shanwu Lyu, Yujuan Liu, Ming Luo, Suhua Shi, Shulin Deng

**Affiliations:** 1 Guangdong Provincial Key Laboratory of Applied Botany & CAS Key Laboratory of South China Agricultural Plant Molecular Analysis and Genetic Improvement, South China Botanical Garden, Chinese Academy of Sciences, Guangzhou 510650, China; lishun@scbg.ac.cn (S.L.); shanwu.lyu@scbg.ac.cn (S.L.); luoming@scbg.ac.cn (M.L.); 2 School of Life Sciences, University of Chinese Academy of Sciences, Beijing 100049, China; liuyujuan@scbg.ac.cn; 3 Center of Economic Botany, Core Botanical Gardens, Chinese Academy of Sciences, Guangzhou 510650, China; 4 State Key Laboratory of Biocontrol, Guangdong Provincial Key Laboratory of Plant Resources, School of Life Sciences, Sun Yat-Sen University, Guangzhou 510275, China; lssssh@mail.sysu.edu.cn; 5 National Engineering Research Center of Navel Orange, School of Life Sciences, Gannan Normal University, Ganzhou 341000, China

**Keywords:** *Cauliflower mosaic virus*, P6, HD2C, biotic stress, ABA

## Abstract

Histone deacetylases (HDACs) are vital epigenetic modifiers not only in regulating plant development but also in abiotic- and biotic-stress responses. Though to date, the functions of HD2C—an HD2-type HDAC—In plant development and abiotic stress have been intensively explored, its function in biotic stress remains unknown. In this study, we have identified HD2C as an interaction partner of the *Cauliflower mosaic virus* (CaMV) P6 protein. It functions as a positive regulator in defending against CaMV infection. The *hd2c* mutants show enhanced susceptibility to CaMV infection. In support, the accumulation of viral DNA, viral transcripts, and the deposition of histone acetylation on the viral minichromosomes are increased in *hd2c* mutants. P6 interferes with the interaction between HD2C and HDA6, and P6 overexpression lines have similar phenotypes with *hd2c* mutants. In further investigations, P6 overexpression lines, together with CaMV infection plants, are more sensitive to ABA and NaCl with a concomitant increasing expression of ABA/NaCl-regulated genes. Moreover, the global levels of histone acetylation are increased in P6 overexpression lines and CaMV infection plants. Collectively, our results suggest that P6 dysfunctions histone deacetylase HD2C by physical interaction to promote CaMV infection.

## 1. Introduction

Acetylation of nucleosomal core histones usually reduces the affinity for DNA, giving an “open” chromatin configuration that allows the combination of transcription factors for transcriptional activation. In contrast, histone deacetylation usually gives rise to a “closed” compact chromatin that alienates transcription factors, resulting in transcriptional repression [1,2,3]. Histone acetylation is catalyzed by histone acetyltransferases (HATs), and deacetylation is catalyzed by histone deacetylases (HDACs). Plant HDACs have been divided into four types, of which three (RPD3, HDA1, and SIR2 types) are homologous to yeast HDACs. The fourth type HDACs, HD2-type deacetylases—first identified in maize—are plant-specific [4,5]. HD2-type deacetylases are homologous to FKBP-type peptidyl-prolyl cis-trans isomerases (PPIases), which can physically associate with RPD3-type HDACs [6,7].

HD2C is one of four HD2 proteins identified in Arabidopsis besides HD2A, HD2B, and HD2D [5,8,9]. AtHD2C has been reported to function as a transcriptional repressor and repress *FT* expression through its deacetylation activity by interacting with H3K4/H3K36 methylation reader MRG2 [10,11]. AtHD2C is required for ribosome biogenesis through canonically repressing genes involved in ribosome biogenesis and noncanonically promoting pre-RNA processing by interacting with HD2B [12]. AtHD2C enhances early germination of Arabidopsis in the HXK1-independent pathway and is needed for leaf and root development [12,13]. Besides development, AtHD2C also participates in abiotic stress [14]. AtHD2C overexpression plants showed high tolerance to salt and drought [15]. It is also associated with RPD3-type HDACs, HDA6, and HDA19, and responds to ABA and salt-stress, with *hd2c* and *hd2c/hda6* exhibiting hypersensitivity to ABA and NaCl [16,17,18]. Buszewicz et al., (2016) reported that HD2C was up-regulated in heat-stressed Arabidopsis. In response to heat stress, HD2C was proved to regulate heat-responsive genes by physically cooperating with chromatin remodeling complex like SWI/SNF [19]. HD2C had also been identified as a core factor in chromatin structure switching from repressed to active status in response to cold stress, whose degradation was facilitated by interacting with HOS15, a substrate receptor of CULLIN4 (CUL4) -based E3 ubiquitin ligase complex, under cold stress [20,21,22]. Although the function of HD2C in abiotic stress was extensively studied, its functions in biotic stress have remained unclear.

HDACs have been reported to participate in plant immunity in response to biotic stress. Latrasse and colleagues found that AtHD2B can reprogram the chromatin landscape of biotic stress response genes to mediate plant innate immunity. They also found that MAP kinase MPK3 was physically associated with phosphorylated HD2B, resulting in the relocation of HD2B from the nucleolus to the nucleoplasm and regulating the expression of flg22-regulated genes by modifying H3K9ac [23]. OsHDT701, an HD2-type HDAC, could regulate the expression of defense-related genes to negatively regulate the defense to *Magnaporthe oryzae* in rice [24]. HDA19, an RPD3-type HDAC, has previously been reported to be induced by *Alternaria brassicicola*, *Pseudomonas syringae*, and defense-related hormones (jasmonic acid and ethylene) [25,26]. Overexpression of HDA19 in Arabidopsis showed increased resistance to *A. brassicicola* and *P. syringae*. Overexpression of AtSRT2, an SIR2-type HDAC, elicited hypersensitivity to *PstDC3000* infection, with reduced expressions of pathogen-related gene PR1 and SA biosynthesis-related genes, indicating a negative function of AtSRT2 in plant innate immunity [27]. Although HDACs have been reported to function in plant immunity in response to fungal and bacterial invasion, their roles in plant viral defense remain poorly explored. Previously, a report demonstrated that the transcriptional gene silencing suppressor V2 of *Tomato yellow leaf curl virus* (TYLCV) competitively interacted with NbHDA6 against methyltransferase 1 (MET1) to reduce DNA methylation of the viral genome [28]. A homolog of histone deacetylase AtSRT2 in tobacco, SIP428, has been confirmed to be down-regulated in response to *Tobacco mosaic virus* (TMV) infection [29]. More recently, a list of TaHDACs have been identified to be up-regulated in response to RNA viruses *Barley stripe mosaic virus* (BSMV), *Wheat yellow mosaic virus* (WYMV), and *Chinese wheat mosaic virus* (CWMV) infection, and one of them, TaSRT1, acted as a negative regulator for defense during CWMV infection [30]. Studies concerning the function of HDACs in plant viral defense are limited, and there is no research about the function of HD2C in DNA viral defense.

*Cauliflower mosaic virus* (CaMV) is a DNA pararetrovirus with a double-stranded DNA genome and causes heavy agricultural losses in crops like cauliflower, broccoli, and turnip. In hosts, the double-stranded DNA genome is associated with host histones to form minichromosomes [31,32], which is crucial for the mutual competition between hosts and CaMV. Recently, a report demonstrated that there is a high level of methylation on the CaMV genome in the nucleus, which is a plant defense strategy to inhibiting virus reproduction [33]. Research has been reported that chromatin modification of the *Cabbage leaf curl virus* (CaLCuV) minichromosome is a major target for the interaction between virus and host. For example, H3K9 methylation associated with gene silencing and H3K4 methylation and H3 acetylation associated with gene expression are both associated with CaLCuV minichromosome [34]. The P6 protein of CaMV has multiple impacts on the interaction between CaMV and host, including affecting host defense signaling. The analysis of P6 overexpression lines showed that P6 could suppress the expression of SA-responsive genes like *BGL2* and *PR1* and enhance the expression of JA-responsive genes, resulting in greatly enhanced susceptibility to *P. syringae* and reduced susceptibility to *Botrytis cinerea* [35]. The expression of SA/JA crosstalk regulator *NPR1* was also up-regulated, and it was re-localized from cytoplasm to nucleus in P6 overexpression lines even without treatment with SA. In addition, suppressor mutants of P6 showed reduced susceptibility to CaMV infection and ethylene. Moreover, two ethylene response mutants, *etr1-1* and *ein2-1*, exhibited decreased susceptibility to CaMV infection [36,37]. In this scenario, whether P6 can interfere with chromatin modification of minichromosome against host defenses through defense-related hormone pathways deserves further investigation.

This study investigated how the multifunctional protein CaMV P6 interfered with the deposition of histone acetylation on viral minichromosome through dysfunction AtHD2C against host defenses. We found that CaMV P6 interacted with HD2C and interfered with the interaction between HD2C and HDA6. Moreover, HD2C acted as a positive defense regulator during the CaMV infection by regulating the level of histone acetylation on the viral minichromosome. In addition, P6 overexpression lines were hypersensitive to the ABA and NaCl, which was similar to that of the *hd2c* mutants. Furthermore, overexpressing P6 or CaMV infection altered the expression and histone modification of ABA-regulated genes, *ABI1*, *ABI2*, and *ERF4*. Our studies provide a foundation for the function of HD2C in CaMV infection.

## 2. Materials and Methods

### 2.1. Plants and Virus Inoculation

Wild type *Arabidopsis thaliana* (Col-0) and two published null HD2C mutant alleles, *hd2c-1* (Salk_129799) and *hd2c-3* (Salk_039784), were used in this study. Arabidopsis seeds were sown on 1/2 Murashigeand Skoog (MS) medium plates. One-week-old germinated seedlings were transferred into pots with soil and grown in a growth chamber with short-day conditions (8 h light/16 h dark at 22 °C). *Nicotiana benthamiana* plants at the stage with 4-6 leaves were used for bimolecular fluorescence complementation assay and co-immunoprecipitation assays. The CaMV strain, CM1841, was used in this study. CM1841 DNA in plasmid pCa122 was infectious in Arabidopsis leaves as described previously [38,39]. 0.1 g of the leaves with systemic symptoms were collected and ground in 1 mL of phosphate buffer (0.05 M, pH 7.2) to obtain CaMV-infected leaf extract. The CaMV-infected leaf extract solution was then used to inoculate the 8-leaf stage Arabidopsis by rubbing the leaves with carborundum. The control group was mock-inoculated in a similar way using phosphate buffer with non-infected leaf extract to create similar mechanical damage.

### 2.2. Yeast Two-Hybrid Assay

For yeast two-hybrid assay, full-length P6 and HD2C CDS were cloned into pGBKT7 and pGADT7 vectors, respectively, and then the recombinant plasmids were co-transformed into AH109 yeast cells. The combinations of pGBKT7 and pGADT7-HD2C or pGBKT7-P6 and pGADT7 were used as negative controls. The transformants were plated on SD/-Leu/-Trp medium and SD/-Leu/-Trp/-His medium with 5 mM 3-Amino-1,2,4-triazole.

### 2.3. Bimolecular Fluorescence Complementation (BiFC) Assay

BiFC experiments were carried out as described previously [40]. Vectors contained nEYFP-P6 and HD2C-cEYFP were introduced into the GV3101 *Agrobacterium Tumefaciens* strain individually. *Agrobacterium* cells were resuspended to OD 0.6 with infiltration buffer (10 mM MgCl_2_, 10 mM MES, 150 μM acetosyringone). The *Agrobacterium* cell solution was injected into the lower epidermis of *N. benthamiana* leaves using a 1 mL injection syringe. After 2 days of cultivation, the lower epidermis of *N. benthamiana* leaves was peeled off and observed for YFP fluorescence signal using confocal microscopy.

### 2.4. Pull-Down Assay

The full-length P6 and HD2C CDS were cloned into pMAL-2c and pGEX-4T-1 vectors, respectively, and then introduced into *E. coli* strain (Rosetta) to express MBP-P6 and GST-HD2C fusion proteins. The MBP- and GST-fusion proteins were induced by 0.2 mM IPTG at 18 °C for 20 h. For protein purification, cells were centrifuged and resuspended using lysis buffer (20 mM Tris-HCl pH 7.5, 500 mM NaCl, 1 mM EDTA, 10 mM β-mercaptoethanol, 10% glycerol for the MBP-fusion protein and 50 mM Tris-HCl pH 7.5, 100 mM NaCl, 1 mM EDTA, 10% glycerol, 1 mg/mL lysozyme, 1% Triton X-100 for the GST-fusion protein). After sonication, the MBP-P6 and GST-HD2C fusion proteins were purified using MBP beads (NEB, Cat No. E8021S) and GST beads (Cat No. 17-0756-01, GE Healthcare, Chicago, IL, USA) respectively by rotating at 4 °C. The pull-down assay was performed as previously described [41]. The purified MBP-P6 (2 μg) and GST-HD2C proteins (2 μg) were mixed and incubated in a pull-down buffer (PBS, 1% Triton X-100) for 2 h at 4 °C. Then the GST beads were added into the mixture and incubated for an additional 2 h. After incubation, beads were washed six times, and the pulled-down proteins were detected by western blotting.

### 2.5. Co-Immunoprecipitation (Co-IP) Assay

Vectors containing 35s-MYC-P6, 35s-YFP-P6, 35s-MYC-HDA6, 35s-FLAG-HD2C, 35s-YFP, and 35s-FLAG-GFP were transferred into GV3101 *A. Tumefaciens* strain, and then *Agrobacterium* cells were injected into tobacco leaves. After 2 days of incubation, protein extracts were obtained by lysing the tobacco leaves using co-immunoprecipitation buffer (50 mM Tris-HCl, pH 7.5, 150 mM NaCl, 2 mM EDTA, 0.5% Triton X-100, 0.5 mM PMSF, 10% glycerol, 10 mM DTT and protease inhibitor cocktail: Roche, Cat No. 11873580001). Then protein extracts were incubated with anti-FLAG M2 affinity gel (Sigma, St. Louis, MI, USA, Cat No. A2220) for 2 h by rotating at 4 °C. After washing with co-immunoprecipitation buffer, the co-immunoprecipitated proteins were analyzed by western blotting using anti-FLAG (MBL, Cat No. M185-7), anti-GFP (Santa Cruz, CA, USA, Cat No. SC-9996), and anti-MYC (Santa Cruz, Cat No. GTX20032) antibodies.

### 2.6. Detection of Histone H3 Modifications

Total proteins were extracted using extraction buffer (50 mM Tris-HCl, pH 7.5, 150 mM NaCl, 2 mM EDTA, 0.5% Triton X-100, 0.5 mM PMSF, 10% glycerol, 10 mM DTT and protease inhibitor cocktail). The protein samples were separated by 12% SDS-PAGE gels. After separation by electrophoresis, proteins were transferred onto the PVDF membrane and detected using the primary antibodies anti-H3K9K14ac (Millipore, Cat No. 06-599), anti-H3K4me^3^ (AbCam Cat No. ab8580), and anti-Actin (Abbkine Cat No. A01050). Quantification of the band intensities on the western blot was performed using the Image J software.

### 2.7. Treatments and Germination Rates

Sterile Arabidopsis seeds were sown on a 1/2 Murashigeand Skoog (MS) medium containing different concentrations of ABA and NaCl. After 3-day stratification, the seeds were germinated in a long-day condition growth chamber. Germination rates were measured after 2 days, with a criterion that radicle protrudes from episperm. To measure the leaf survival rate of seedlings under high salinity, we transferred 5-day-old seedlings to the medium containing 150 mM NaCl. After 5 days of treatment, the leaf survival rate of seedlings was measured.

### 2.8. RNA Extraction and Quantitative Real-Time PCR

RNA was extracted using the Trizol (RNAiso Plus, TaKaRa Cat No. 9108), and DNA contamination was removed using RQ1 RNase-Free DNase (Promega Cat No. M6101). The first-strand cDNA was synthesized using the GoScript^TM^ Reverse transcription kit (Promega Cat No. A2709). Real-time PCR was performed using qPCR Master Mix (Promega Cat No. LS2062) in a LightCycler 480 thermocycler (Roche, Basel, Switzerland). The gene-specific primer sequences are listed in Supplemental Table S1.

### 2.9. Chromatin Immunoprecipitation (ChIP)

The ChIP assay was performed as described previously with slight modification [42]. 2 g Arabidopsis leaves with systemic symptoms were collected three days after the onset of the disease, or 2 g Arabidopsis seedlings were collected from 18-day-old Arabidopsis. All samples were powdered in liquid nitrogen and incubated in M1 buffer (10 mM phosphate buffer pH 7.2, 0.1 M NaCl, 10 mM β-mercaptoethanol, 1 M hexylene glycol) with 1% formaldehyde for 5 min cross-linking, and then stopped the cross-linking reaction by adding Gly to a final concentration of 0.125 M and incubated for 5 min. After incubation, the mixture was filtrated using a four-layer Miracloth and centrifuged. The pellet was suspended, centrifuged, and washed three times using M2 buffer (10 mM phosphate buffer pH 7.2, 0.1 M NaCl, 10 mM β-mercaptoethanol, 1 M hexylene glycol, 10 mM MaCl_2_, 0.5% Triton X-100), and one-time using M3 buffer (10 mM phosphate buffer pH 7.2, 0.1 M NaCl, 10 mM β-mercaptoethanol). The nuclei pellet was suspended using nuclei lysis buffer (50 mM Tris-HCl pH8, 10 mM EDTA, 1% SDS) and sonicated to fragment the chromosomal DNA. Immunoprecipitation was performed using an anti-H3K9K14ac (Millipore, Cat No. 06-599) antibody attached with salmon sperm DNA/protein A agarose beads (all samples used the same volume of antibodies and beads). The beads were successively washed with low-salt, high-salt, LiCl, and TE buffer (Low-salt buffer: 150 mM NaCl, 0.1% SDS, 1% Triton X-100, 20 mM Tris-HCl pH8, 2 mM EDTA; High-salt buffer: 500 mM NaCl, 0.1% SDS, 1% Triton X-100, 20 mM Tris-HCl pH 8, 2 mM EDTA; LiCl buffer: 0.25 M LiCl, 1% Nonidet-P40, 1% sodium deoxycholate, 1 mM EDTA; TE buffer: 10 mM Tris-HCl pH 8, 1 mM EDTA) one time for 5 min. The protein-DNA complex was eluted using elution buffer (1% SDS, 0.1 M NaHCO_3_) and then take 30 μL of elution product to detect the precipitated protein using anti-H3K9K14ac antibody by western blot to ensure the efficiency of precipitation. The remaining elution product was incubated at 65 °C overnight to reverse cross-linked. After removing protein by adding proteinase K and incubating for 1 h at 45 °C, the DNA was precipitated by adding sodium acetate (pH 5.2), glycogen, and ethanol. The purified DNA was then used for real-time PCR, and the primers are listed in Supplementary Table S2. *ACTIN7* was used for normalization.

## 3. Results

### 3.1. CaMV P6 Interacts with Histone Acetyltransferase AtHD2C in Nucleoli

**Figure 1:**
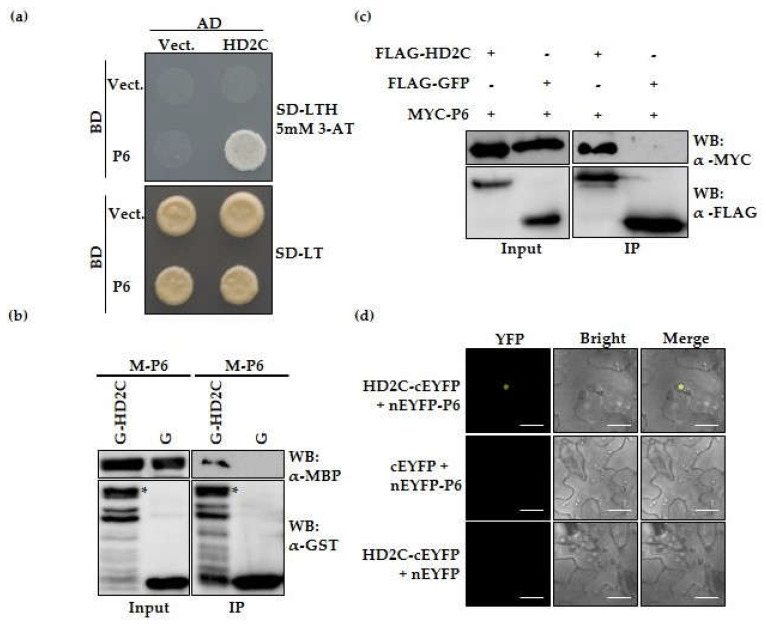
P6 interacts with HD2C in vivo and in vitro. (**a**) Yeast two-hybrid assays were performed by expressing BD-P6 and AD-HD2C fusion proteins in yeast cells. Interaction of BD-P6 and AD-HD2C was examined by growing yeast cells on SD–LTH selective medium supplement with 5 mM 3-Amino-1, 2, 4-triazole. SD-LTH indicates Leu, Trp, and His drop-out medium. SD-LT indicates Leu and Trp drop-out medium. (**b**) Pull-down assays. The purified MBP-P6 fusion protein was incubated with GST or GST-HD2C protein. The co-precipitation of MBP-P6 by GST-HD2C was detected using the anti-MBP antibody. GST fusion proteins were detected using the anti-GST antibody. Asterisk (*) indicates the recombinant target protein. M: MBP, G: GST. (**c**) In vivo co-immunoprecipitation assays were performed by co-expressing FLAG-HD2C or FALG-GFP with MYC-P6 fusion protein in *N. benthamiana*. FALG-GFP was used as a negative control. Crude extracts (Input) and immunoprecipitates (IP) were detected using anti-FLAG and anti-MYC antibodies. (**d**) BiFC assay. P6 fused with nEYFP and HD2C fused with cEYFP were transiently co-expressed in *N. benthamiana*. The YFP fluorescence signal was observed on confocal microscopy. Bars, 20 μm.

To seek host proteins related to CaMV infection, we previously performed an affinity purification-mass spectrometry using transgenic Arabidopsis 35S: FLAG-P6 and control transgenic Arabidopsis 35S: FLAG-EGFP to identify possible interaction partners of P6 by an anti-FLAG antibody. The histone acetyltransferase AtHD2C was found to co-purify with P6. AtHD2C is located in nucleoli, involved in rRNA maturation and ribosome biogenesis [12]. To verify the previous affinity purification-mass spectrometry assay results, we cloned full-length HD2C and P6 CDS into pGADT7 and pGBKT7 vectors, respectively, and the yeast two-hybrid assay was performed to test the interaction between P6 and AtHD2C. The resulting transformants grew on a selective medium with 5 mM 3-Amino-1, 2, 4-triazole indicated that HD2C interacted with P6 (Figure 1a). We also conducted in vitro pull-down analysis. As shown in Figure 1b, full-length P6 fused with MBP tag was pulled down by full-length AtHD2C fused with GST, but not by GST alone. To further prove the interaction in vivo, we carried out co-immunoprecipitation (Co-IP) and bimolecular fluorescence complementation (BiFC) assays. Co-IP experiments revealed that MYC-P6 was co-precipitated with FLAG-HD2C but not with the negative control, FLAG-GFP, in *N. Benthamiana* (Figure 1c). In addition, co-expression of HD2C-nEYFP and cEYFP-P6 yielded an unmistakable BiFC fluorescence signal in nucleoli (Figure 1d), which was in line with the localization of HD2C [4,12]. These results elucidated that HD2C has a specific and direct interaction with CaMV P6 in vitro and in vivo.

### 3.2. Mutants of AtHD2C Are More Susceptible to CaMV Infection

**Figure 2:**
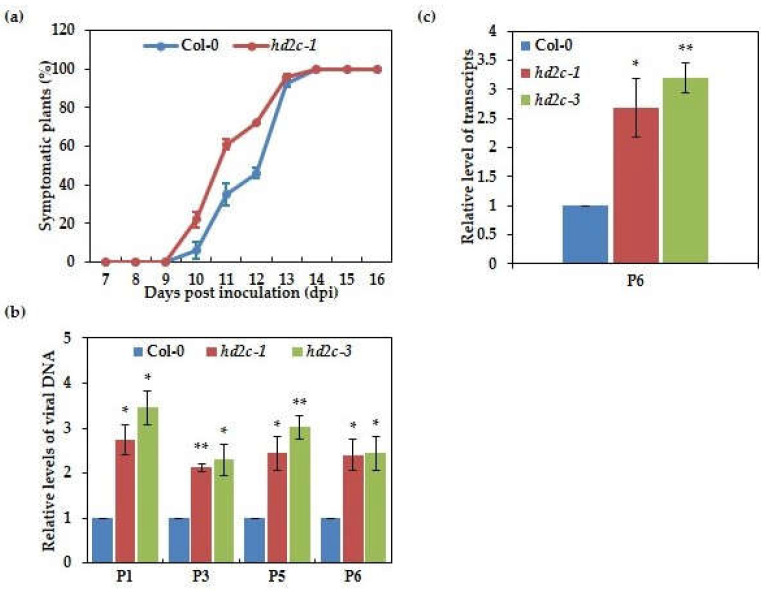
Mutants of AtHD2C are more susceptible to CaMV infection. (**a**) The susceptibility of wild-type and *hd2c-1* mutant to CaMV. Symptomatic plants were measured and shown as percentages at different days after infection. (**b**) The accumulation of CaMV DNA (P1, P3, P5, and P6) in CaMV infected wild-type and *hd2c* mutants at 8 dpi. P1, P3, P5, and P6 specific primers were used in qPCR, and *ACTIN2* was used as an internal control. Values significantly different from the wild-type were marked using asterisks (*t*-test, ** *p* < 0.01, * *p* < 0.05). (**c**) Relative accumulation of CaMV P6 transcript from 8 dpi CaMV infected wild-type and *hd2c* mutants. P6 specific primer was used in qPCR, and *ACTIN2* was used as an internal control. The statistical significance was determined by performing a *t*-test (** *p* < 0.01, * *p* < 0.05).

It is reported that a histone H4 deacetylase, OsHDT70, could regulate the level of histone H4 acetylation on the promoter region of defense-related genes to negatively regulate innate immunity in rice, providing insight into the roles of histone deacetylase in biotic stress responses [24]. More recently, a study has shown that TaHDACs were involved in the response to RNA viral infections, with increased expression during RNA viral infections in *Triticum aestivum* [30]. In order to investigate the biological significance of the P6-AtHD2C interaction in plant DNA viral infection, we first attempted to test whether HD2C affects CaMV infection in Arabidopsis using *hd2c-1*, a mutant of HD2C. We carried out a viral inoculation experiment using the wild-type (Col-0) as a control and recorded symptom development. As shown in Figure 2a, although the symptoms and severity of the infected Col-0 and *hd2c-1* plants were similar, both exhibited chlorosis, vein-clearing and leaf mosaics, half of the *hd2c-1* mutant plants showed disease symptoms at 10.5 days post-inoculation (dpi), while half of the Col-0 plants showed disease symptoms at 12 dpi with a delay of 1.5 days. In the further experiment, viral DNA and transcript accumulation were tested by qPCR using P1, P3, P5, and P6 specific primers. As shown in Figure 2b,c, viral DNA and P6 transcript at 8 dpi accumulated at greater levels in HD2C mutants than Col-0 plants. These results suggest that HD2C functions as a positive regulator in resistance to CaMV infection.

### 3.3. AtHD2C Functions to Inhibit the Viral Gene Expression on Minichromosome through Histone Deacetylation

**Figure 3:**
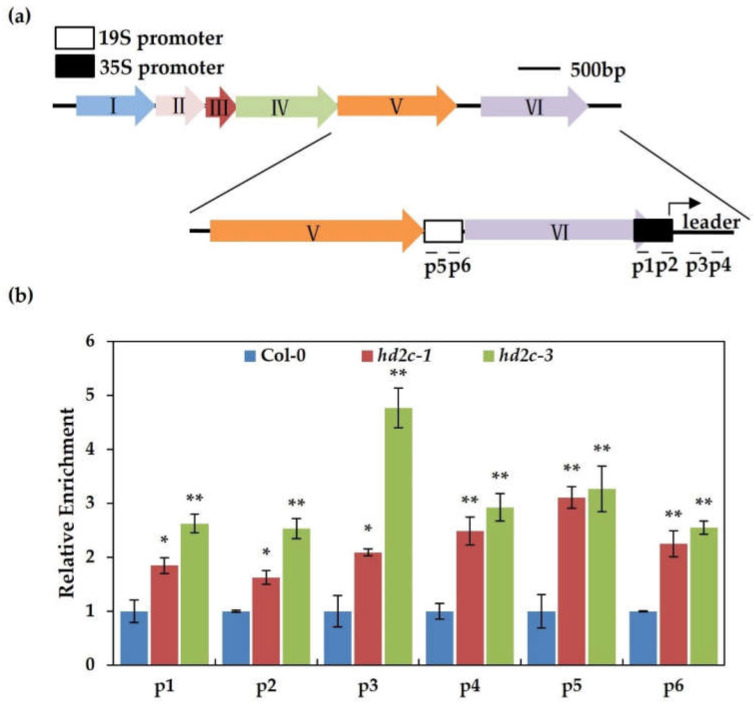
AtHD2C inhibits the viral gene expression on minichromosomes through histone deacetylation. (**a**) Schematic diagram of 19S and 35S promoters of CaMV. The p1-p6 sequence regions were chosen for ChIP-qPCR assays. (**b**) Relative enrichment of H3K9K14ac on CaMV minichromosome. qPCR was performed to quantify the amounts of DNA obtained from ChIP, and *ACTIN7* was used as an internal control. Values significantly different from the wild-type were marked using asterisks (*t*-test, ** *p* < 0.01, * *p* < 0.05). The experiment was repeated three times with similar results.

In order to propagate in the host, plant DNA viruses like geminiviruses and CaMV use host histone to pack their DNA genome into a chromatin-like structure called minichromosome [32,43]. Furthermore, it has been proven that both chromatin-activation modification and chromatin-repressive modification are present in geminivirus minichromosome [44]. On account of the genome of CaMV, which can be packed by host histone into minichromosome after infection, we tested whether AtHD2C could influence the viral gene expression on minichromosome through histone deacetylation. We conducted a chromatin immunoprecipitation and qPCR to test the level of histone acetylation on the CaMV minichromosome. As shown in Figure 3a,b, the depositions of histone acetylation on 35S and 19S promoter regions of viral minichromosome were significantly increased in *hd2c* mutants than the wild-type, suggesting that AtHD2C may have a role to inhibit the viral gene expression on minichromosome through histone deacetylation.

### 3.4. CaMV Infection and Overexpression of P6 Dysfunction AtHD2C

**Figure 4:**
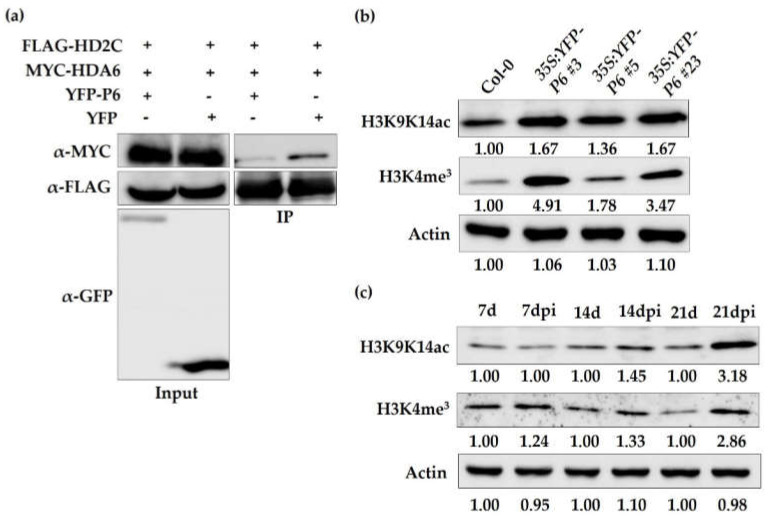
P6 dysfunctions HD2C. (**a**) P6 competitively interacted with HD2C against HDA6. The fusion proteins FLAG-HD2C and MYC-HDA6 were co-expressed with YFP-P6 or YFP in the same *N. benthamiana* leaf. After immunoprecipitation, fusion proteins were detected using anti-FLAG, anti-MYC, and anti-GFP antibodies by western blot. (**b**) The levels of histone H3K9K14ac and H3K4me^3^ in wild-type and P6 overexpression lines. The loading control immunoblots of Actin were used for normalization. (**c**) The levels of histone H3K9K14ac and H3K4me^3^ in CaMV infected (7 dpi, 14 dpi, and 21 dpi) and non-infected (7 d, 14 d, and 21 d) plants. The loading control immunoblots were shown as the levels of Actin. dpi: days post-inoculation.

Given that HD2C had been previously reported to interact with HDA6 [16], we investigated whether P6 competitively interacted with HD2C against HDA6. Indeed, P6 interfered with the interaction between HD2C and HDA6, considering the amount of HDA6 co-precipitated with HD2C decreased when co-expressed with P6 (Figure 4a). As histone deacetylase, HD2C and HDA6 function to remove acetylation modification of histone H3, with globally increased levels of H3K9K14ac and H3K4me^3^ in *hd2c-1* and *hda6* mutants [16]. To explore the influence of the interaction between P6 and HD2C to H3K9K14ac and H3K4me^3^ global levels, we analyzed the levels of H3K9K14ac and H3K4me^3^ in P6 overexpression lines and CaMV infected plants. Our results have shown that the levels of H3K9K14ac and H3K4me^3^ are both globally increased in P6 overexpression lines compared with the wild-type (Figure 4b). Similar results were also observed in CaMV infected plants compared with control (Figure 4c). With the lastingness of infection, the amount of viral minichromosome increased, and the levels of H3K9K14ac and H3K4me^3^ increased gradually (Figure 4c). These results demonstrated that P6 interfered with the function of HD2C by physical interaction, and the underlying mechanism of P6 promoting CaMV infection in the wild was to impede HD2C to reducing the level of H3K 9K14ac on viral minichromosome.

### 3.5. Overexpression of P6 Increases Sensitivity to ABA and NaCl

**Figure 5:**
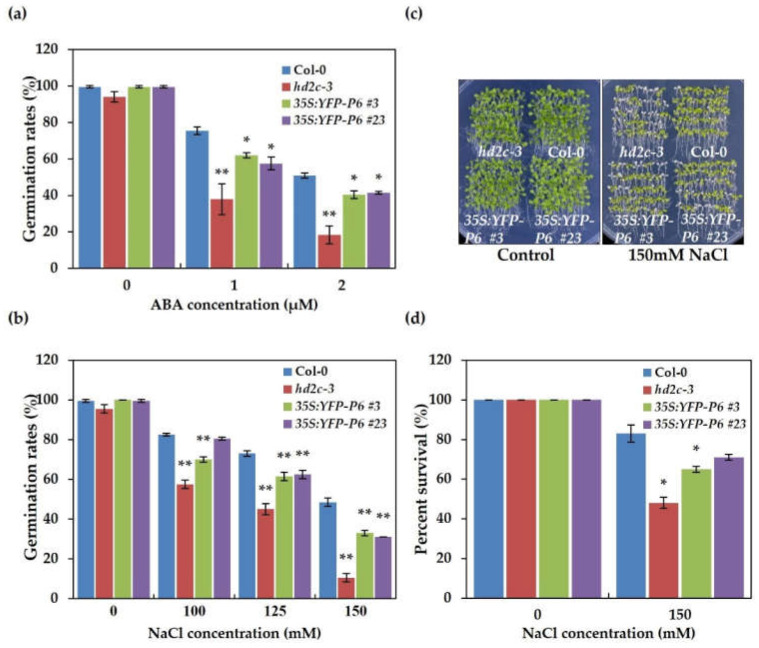
Overexpression of P6 increased sensitivity to ABA and NaCl stress similar to *hd2c* mutants. (**a**) Seed germination rates of wild-type, *hd2c-3*, and P6 overexpression lines treated with ABA. Standard deviation was derived from three independent experiments (*n* ≥ 100). Values that are significantly different from the wild-type were marked using asterisks (*t*-test, ** *p* < 0.01, * *p* < 0.05). (**b**) Seed germination rates of wild-type, *hd2c-3*, and P6 overexpression lines treated with NaCl. Standard deviation was derived from three independent experiments (*n* ≥ 100). Values that are significantly different from the wild-type were marked using asterisks (*t*-test, ** *p* < 0.01, * *p* < 0.05). (**c**) Phenotypes of P6 overexpression lines, *hd2c-3*, and wild-type in response to salt stress. Five-day-old seedlings were transferred to the medium containing 150 mM NaCl. (**d**) The leaf survival rate of wild-type, P6 overexpression lines, and *hd2c-3* seedlings. Standard deviation was derived from three independent experiments (*n* = 50). Values that are significantly different from the wild-type were marked using asterisks (*t*-test, * *p* < 0.05).

If P6 surely dysfunction HD2C, we speculated that some phenotypes of P6 overexpression lines might be similar to that of HD2C mutants. AtHD2C has had reports that state that ABA and NaCl can repress its expression [15]. Two HD2C mutants were supersensitive to ABA and NaCl, whose seed germination rate decreased when treated with ABA and NaCl [16]. To confirm our hypothesis and study the effect of biotic stress caused by the CaMV virus on the abiotic stress, we investigated the seed germination rate of P6 overexpression lines under the ABA and NaCl stress. Overexpressing P6 showed similar phenotypes to *hd2c-3*, with lower germination rates than the wild-type under the ABA and NaCl stress (Figure 5a,b). We also investigated the leaf survival rates of P6 overexpression lines under high salinity stress. Five-day-old Arabidopsis seedlings of P6 overexpression lines, *hd2c-3*, and wild-type were transferred to 1/2 MS medium containing 150 mM NaCl for 5 days. Leaves left green were measured as the percentage of leaf surviving. The leaf survival rates of P6 overexpression lines were lower than the wild-type, similar to *hd2c-3* (Figure 5c,d). These results indicated that P6 overexpression lines were more sensitive to ABA and NaCl, and functional defects of HD2C might endow this.

### 3.6. Histone H3K9K14ac Levels of ABI1 and ABI2 Are Changed in P6 Overexpression Lines

**Figure 6:**
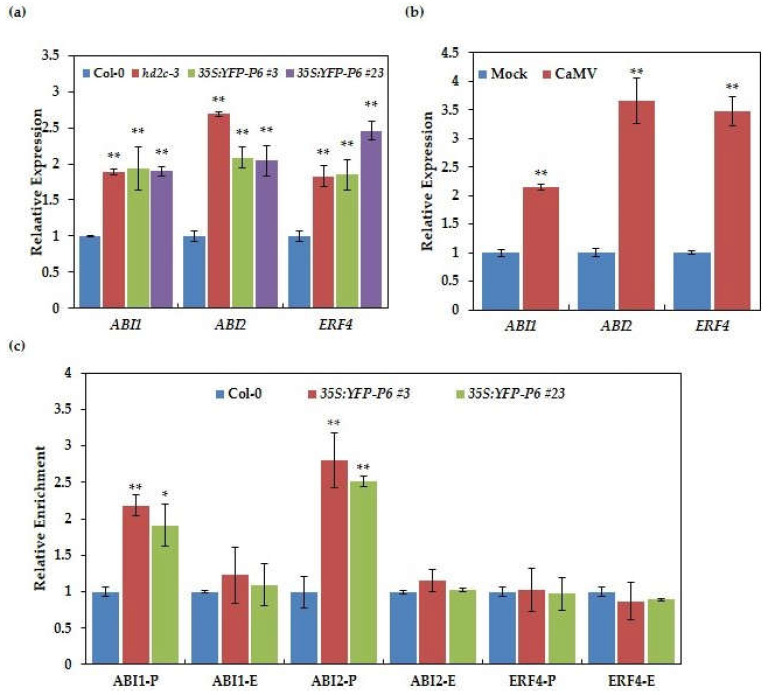
Overexpression of P6 enhances the expression and the deposition of H3K9K14ac of ABA-regulated genes. (**a**) The relative expression of *ABI1*, *ABI2*, and *ERF4* in P6 overexpression lines. Total RNA was extracted from 14-day-old seedlings, and qPCR was performed using *ABI1*, *ABI2*, and *ERF4* specific primers. Values that are significantly different from the wild-type were marked using asterisks (*t*-test, ** *p* < 0.01). The experiment was repeated three times with similar results. (**b**) The relative expression of *ABI1*, *ABI2*, and *ERF4* in CaMV infected and non-infected (Mock) plants at 14 dpi. Total RNA was extracted from leaves with systemic symptoms. The experiment was repeated three times with similar results. (**c**) Relative enrichment of H3K9K14ac on *ABI1*, *ABI2*, and *ERF4* in wild-type and P6 overexpression lines. qPCR was performed to quantify the amounts of DNA obtained from ChIP, and *ACTIN7* was used as an internal control. P: the promoter, E: the first exon. Values that are significantly different from the wild-type were marked using asterisks (*t*-test, ** *p* < 0.01, * *p* < 0.05). The experiment was repeated three times with similar results.

Luo et al., (2012) showed that HD2C and HDA6 influenced the H3K9K14 acetylation level on the promoter region of ABA-responsive genes, *ABI1* and *ABI2*, to regulate gene expression [16]. To explore the underlying molecular mechanism of P6 overexpression lines displaying hypersensitivity to ABA and NaCl and further confirm that P6 dysfunction HD2C, we detected the expression of *ABI1*, *ABI2*, and *ERF4* in P6 overexpression lines. The qPCR results showed that the expressions of *ABI1*, *ABI2*, and *ERF4*, were increased in *hd2c* mutant and elevated in the P6 overexpression lines than the wild-type (Figure 6a). We further detected the expression of these ABA-regulated genes in CaMV infected plants compared with non-infected plants. As shown in Figure 6b, the expression of these ABA-regulated genes were both increased in CaMV infected plants compared with non-infected plants, suggesting that CaMV infection altered the expression pattern of ABA signaling genes. To verify these results, we conducted a chromatin immunoprecipitation and qPCR to detect the deposition of H3K9K14ac on these ABA-regulated genes. ChIP-qPCR result showed that the deposition of H3K9K14ac in the promoter regions of *ABI1* and *ABI2* in P6 overexpression lines was higher than wild-type. While there is no significant difference in the deposition of H3K9K14ac at *ERF4*. These results indicated that the underlying molecular mechanism of P6 overexpression lines displaying hypersensitivity to ABA and NaCl may be that P6 regulated the expression of *ABI1* and *ABI2* by dysfunction HD2C (Figure 6c).

## 4. Discussion

### 4.1. Histone Acetylation Participates in Biotic Stress Responses against DNA Viral Infection

AtHD2B and OsHDT701, members of HD2-type HDAC, have been reported to control the expression of biotic stress response genes by directly binding to them and modulating histone acetylation levels, playing vital roles in the host defense against bacteria and fungi pathogens [23,24]. More recently, TaHD2D, an HD2-type HDAC in *Triticum aestivum*, was reported significantly induced by the infection of three RNA viruses like BSMV, CWMV, and WYMV, with a pity that the role of TaHD2D in viral infection and the involved mechanisms were unclear [30]. This report also found other types of TaHDACs like TaHDA6, TaHDA15, TaHDA2, and TaSRT1 involved in the stress response to RNA virus pathogens. Furthermore, the expression of SIP428, an SIR2-type HDAC in tobacco, showed a slight decrease in response to TMV infection [29]. Recently, it was reported that a list of histone acetyltransferases (HAT) showed elevated expression when the inoculation with RNA virus occur in *Triticum aestivum* [45]. Although a previous report had confirmed that histone deacetylase NbHDA6 influenced the infection of TYLCV, a DNA virus, through interaction with methyltransferase 1 (MET1) and TYLCV V2 protein [28], there is no evidence to prove whether histone acetylation and deacetylation regulate the stress response to DNA virus pathogens. In this work, we found that histone deacetylase HD2C functions as a positive regulator in defense response to CaMV infection, and its regulation of histone deacetylation on DNA-viral minichromosome was interfered by interaction with CaMV P6 protein.

### 4.2. CaMV P6 Interacts with HD2C to Interfere with Histone Deacetylation

HD2-type HDACs have a conserved C2H2 type zinc finger domain, which may mediate protein-protein or DNA-protein interactions [8,9]. Previously, HD2C was found to interact with two RPD3-type HDACs, HDA6, and HDA19 in nucleoli [17]. Here, we identified HD2C as a CaMV P6 interacting partner, whose interaction also occurred in nucleoli (Figure 1d). The interaction between viral protein and HDACs had been previously demonstrated. For instance, the transcriptional gene silencing suppressor, TYLCV V2 protein, had been found to interact with HDA6 directly in *N. benthamiana* [28]. This report also demonstrated that V2 protein interfered with the gene-silencing function of HDA6 by impeding the interaction between HDA6 and methyltransferase 1 (MET1). Given that HDA6 co-precipitated with HD2C was decreased when co-expressed with P6 (Figure 4a), we concluded that the interaction between HDA6 and HD2C was interfered with by CaMV P6. This result gave insight into a possibility that P6 causes HD2C to dysfunction, leading us to speculate that the overexpression of P6 might have a similar phenotype with *hd2c* mutants. HD2C and HDA6 have been previously identified as a regulator of ABA, and their mutants exhibited hypersensitivity to ABA and NaCl [15,16,46]. To verify our conjecture, we first detected the global level of H3K9K14ac and H3K4me^3^ in P6 overexpression lines and found that the level of these modifications increased (Figure 4b). Seeds germination and seedling treatment with ABA and NaCl were further performed. Consistent with the hypothesis, seed germination rates and the leaf survival rates of the P6 overexpression lines were lower than the wild-type when treated with ABA and NaCl, similar to *hd2c* mutant (Figure 5). HD2C cooperates with HDA6 as a complex to regulate gene expression through histone deacetylation modification, and a recent report demonstrated that knocking down TaHDT701, a homolog of AtHD2C in *Triticum aestivum*, impaired the TaHDA6 occupancy at the promoters of defense response genes [16,47]. It is, therefore, possible that the dysfunction of HD2C caused by P6 may also influence the function of HDA6, leading to similar phenotypes between overexpression lines and *hda6* mutant. For example, both P6 overexpression lines and *hda6* mutant exhibited late flowering [48,49].

### 4.3. HD2C Functions as a Positive Regulator in Defense Response to DNA Virus Infection

Pathogen-induced histone modification is a hotspot of research. Histone acetylation and deacetylation were involved in host defense responses to virus pathogens [28,29,30,45,50]. HD2C is a well-studied and characterized HDAC, whose function is established not only in plant development but in plant abiotic and biotic stress responses [14]. However, the involvement of HD2C in biotic stress responses was limited to bacterial and fungal pathogens, with unknown functions in antiviral response and the underlying mechanisms. Our results have shown that *hd2c-1* was hypersensitive to CaMV infection, and the amount of viral DNA and the expression of the viral transcript were higher in *hd2c* mutants than the wild-type, indicating a positive regulatory role of HD2C in antiviral response (Figure 2). As a deacetylase, HD2C repressed gene expression by removing the acetyl group from histone. Thus, the underlying mechanism that HD2C positively resisting against CaMV is possible to repress viral gene expression through histone deacetylation on viral minichromosome. Our ChIP results indicated that the levels of histone deacetylation on 35S and 19S promoter regions of viral minichromosome were significantly increased in *hd2c* mutants than in the wild-type (Figure 3b). Together, these results provide a foundation for insight into the effect of HD2C on DNA viral defense. However, our studies could not exclude the possibility that additional HDACs are also involved in this process; after all, HD2C may work together with additional HD2-type or RPD3-type HDACs as a complex with gene regulation activity [14,17].

### 4.4. CaMV Facilitates Host Adaptation under Abiotic Stress by Histone Deacetylation

It is increasingly recognized that viral infections can facilitate host adaptation to abiotic stress, raising a novel idea of using viral infection as a probe to uncover the unknown molecular mechanisms of the abiotic stress response. An example was that virus infection by a list of RNA viruses significantly enhanced plant tolerance to drought and freezing stress, with a probable mechanism that the level of osmoprotectant and antioxidant were increased in infected plants [51]. Further research revealed that the drought tolerance induced by *Cucumber mosaic virus* (CMV), an RNA virus, was caused by the 2b viral protein and the 2b-transgenic plants exhibited hypersensitivity to ABA-mediated inhibition of germination [52]. Another report demonstrated that plants subjected to more viral virulence showed higher tolerance to drought than plants subjected to lesser viral virulence [53]. To finish the life cycle and propagation, viruses like TYLCV facilitated the host to survive heat stress [54,55], which may be a universal phenomenon in the interaction between virus and host. Here, we show that the interaction between CaMV P6 and HD2C confers a possibility that CaMV may facilitate host adaptation under abiotic stress through histone deacetylation. Our result showed that the expressions of ABA-regulated genes like *ABI1*, *ABI2*, and *ERF4* were significantly induced by overexpressing P6 or CaMV infection (Figure 6a,b). Consistently, the deposition of histone acetylation on these genes was increased in the P6 overexpression lines than in the wild-type (Figure 6c). Previous reports had demonstrated that HD2C was involved in many abiotic stresses like salt, heat, and cold stress [19,21,56]. Therefore, further studies are needed to investigate how CaMV facilitates the hosts to survive these stresses through histone deacetylation.

## 5. Conclusions

In conclusion, HD2C functions as a positive regulator in defense response to DNA virus infection. However, physical interaction between CaMV P6 and HD2C interferes with the function of HD2C, which alters the deposition of histone acetylation on viral minichromosome, and promotes the expression of viral genes, and eventually promotes virus reproduction.

## Data Availability

Not applicable.

## Supplementary Materials

The following are available online at https://www.mdpi.com/article/10.3390/cells10092278/s1, Table S1: Primers used for RT-PCR analysis, Table S2: Primers used for ChIP assay.
