# Supplementary material for: Cauliflower mosaic virus P6 Dysfunctions Histone Deacetylase HD2C to Promote Virus Infection
Source: Cells. 2021 Sep 1;10(9):2278. doi: 10.3390/cells10092278 (PMC8464784; doi:10.3390/cells10092278)
Supplement: Supplementary file 1 [file cells-10-02278-s001.zip › cells-1340184-supplementary.pdf]

Supplementary Tables:

Table S1. Primers used for RT-PCR analysis

Genes	Primers
ABI1	F: 5′ – GCCATGTCGAGATCCATTGG – 3′
	R: 5′ – AACGATGCATCCCCAGCCAC – 3′
ABI2	F: 5′ – CAAGATCCATTGGCGATAGATACC – 3′
	R: 5′ – CCTCTTTTCTCCGCCGGAAG – 3′
AtERF4	F: 5′ – CGGCTACTACTAACCAGACCC – 3′
	R: 5′ – TCGCTGAAGGCACAACTAA– 3′
P1	F: 5′ – TTCAAGAAATTGGAAATGTCCAGCA– 3′
	R: 5′ – GGATTGGGTTTTAGCTCCTAATAAGGC– 3′
P3	F: 5′ – GCTTAGAAGCCGTTGCAGCGA– 3′
	R: 5′ – TCCTACGCCGTAATTGGGGTAAG– 3′
P5	F: 5′ – GTGCAATCAACATGGAATCATTCTTTC– 3′
	R: 5′ – TTGAGGCTTGTGTGTTCCTTCATCTAT– 3′
P6	F: 5′ – GGATCTAACAGAACTCGCCGTGAA– 3′
	R: 5′ – GAGTGTCGTGCTCCACCATGTTG– 3′
ACTIN2	F: 5′ – GCCATCCAAGCTGTTCTCTC– 3′
	R: 5′ – GCTCGTAGTCAACAGCAACAA– 3′

Table S2. Primers used for ChIP assay

Genes	Primers
ABI1-P	F: 5′ – GATATTTTACCGGTGGTC – 3′
	R: 5′ – GACGTGTCGTAGTCCGAGTT – 3′
ABI1-E	F: 5′ – CTTGTCTTCCTAGCTTCTTC – 3′
	R: 5′ – CCTTTACCCAATCTGATCCC – 3′
ABI2-P	F: 5′ – CTAGTGTGGTCAGTGTAGATG – 3′
	R: 5′ – GTGTAACATGCCATATGTCAC – 3′
ABI2-E	F: 5′ – CTCTCCTTTCTCTTCCCAAC – 3′
	R: 5′ – GAGGGTCAGTGAATGGTCTG – 3′
AtERF4-P	F: 5′ – GTGTACTCACTAGAGCAAGC – 3′
	R: 5′ – CCGTACAGTATTTGACGC – 3′
AtERF4-E	F: 5′ – ACCATTTCAGGCTTTGAC – 3′
	R: 5′ – AACGCCTCTGTAACGAA– 3′
p1	F: 5′ – GATTCCATTGCCCAGCTATCTGTCA– 3′
	R: 5′ – GCATCTTGAACGATAGCCTTTCCTT– 3′
p2	F: 5′ – GATTGATGTGATATCTCCACTGACGTAAGG– 3′
	R: 5′ – GCGTGTCCTCTCCAAATGAAATGAA– 3′
p3	F: 5′ – GTTTCGCTCACGTGTTGAGCATATAAGAAA– 3′
	R: 5′ – CTGGATTTTAGTACTGGATTTTGGTTTTAGG– 3′
p4	F: 5′ – TAGGCAGGAGGCCGTTAGGGAAAAG– 3′
	R: 5′ – CTCCTTCCTTCCGTCCACTTTATCA– 3′
p5	F: 5′ – GAAACATCAGATGGCAAGCATGGCT– 3′
	R: 5′ – TGAAAGGAAGTCCGCAAAGTGGTTG– 3′
p6	F: 5′ – ACACATCTCTGGAGACTGAGAAAATCAGA– 3′
	R: 5′ – GCTAAGCTTATTTTTGCTCTTACTAGATCGAG– 3′
ACTIN7	F: 5′ – GGAAACATCGTTCTCAGTGGT– 3′
	R: 5′ – CTTGATCTTCATGCTGCTAGGT– 3′
